# Disrupted ‘reflection’ impulsivity in cannabis users but not current or former ecstasy users

**DOI:** 10.1177/0269881108089587

**Published:** 2009-01-01

**Authors:** L Clark, JP Roiser, TW Robbins, BJ Sahakian

**Affiliations:** 1 Behavioural and Clinical Neuroscience Institute, University of Cambridge, Cambridge, UK; 2 Department of Experimental Psychology, University of Cambridge, Cambridge, UK; 3 Institute of Neurology, Queen Square, London, UK; 4 Department of Psychiatry, University of Cambridge, School of Clinical Medicine, Addenbrooke’s Hospital, Cambridge, UK

**Keywords:** addiction, cannabis, decision-making, inhibition, MDMA

## Abstract

Evidence for serotonin involvement in impulsivity has generated interest in the measurement of impulsivity in regular ecstasy users, who are thought to display serotonergic dysfunction. However, current findings are inconsistent. Here, we used a recently developed Information Sampling Test to measure ‘reflection’ impulsivity in 46 current ecstasy users, 14 subjects who used ecstasy in the past, 15 current cannabis users and 19 drug-naïve controls. Despite elevated scores on the Impulsivity subscale of the Eysenck Impulsiveness-Venturesomeness-Empathy questionnaire, the current and previous ecstasy users did not differ significantly from the drug-naive controls on the Information Sampling Test. In contrast, the cannabis users sampled significantly less information on the task, and tolerated a lower level of certainty in their decision-making, in comparison to the drug-naive controls. The effect in cannabis users extends our earlier observations in amphetamine- and opiate-dependent individuals (Clark, *et al*., 2006, *Biological Psychiatry* **60**: 515–522), and suggests that reduced reflection may be a common cognitive style across regular users of a variety of substances. However, the lack of effects in the two ecstasy groups suggests that the relationship between serotonin function, ecstasy use and impulsivity is more complex.

## Introduction

Impaired inhibitory control in drug addiction is thought to underlie a breakdown of self-regulation that causes individuals to continue drug administration, despite growing awareness of the associated negative consequences (Goldstein and Volkow, 2002; Jentsch and Taylor, 1999; Lyvers, 2000). Inhibitory processes can be quantified with neurocognitive measures of impulsivity, where deficient performance has been demonstrated in regular users of a wide range of substances, including stimulants, opiates and alcohol (Bjork, *et al*., 2004; Fillmore and Rush, 2002; Forman, *et al*., 2004). Impulsivity has received particular attention in relation to the regular use of 3,4-methylenedioxymethamphetamine (MDMA) or ‘ecstasy’. Studies of experimental animals have shown that MDMA has selective neurotoxic effects on serotonin (5-hydroxytryptamine, 5-HT) neurons (Gouzoulis-Mayfrank and Daumann, 2006), and this serotonin neurotoxicity may cause or exacerbate impulsivity in human users. There is a longstanding association between reduced serotonin neurotransmission and behavioural impulsivity (Evenden, 1999b; Soubrié, 1986), derived from behavioural pharmacology studies in experimental animals (Tye, *et al*., 1977) and data associating serotonin metabolite and precursor reductions with clinical impulse control disorders (LeMarquand, *et al*., 1999; Linnoila, *et al*., 1983). Consistent with these data, regular ecstasy users were reported to display impulsive responding on several laboratory tests, including the Matching Familiar Figures Test (MFFT) (Morgan, 1998; Morgan, *et al*., 2002; Morgan, *et al*., 2006; Quednow, *et al*., 2007), the Go/No Go test (Moeller, *et al*., 2002b) and the Stroop test (Halpern, *et al*., 2004), in comparison to drug-naïve and polydrug-using control groups.

However, the link between impulsivity and ecstasy use remains problematic. Several case–control designs in MDMA users have failed to replicate a basic deficit on inhibitory measures [Go/No Go (Fox, *et al*., 2002; Gouzoulis-Mayfrank, *et al*., 2003), Stop Signal Test (von Geusau, *et al*., 2004), Stroop test (Dafters, 2006)]. Where positive results have been reported, these effects may be limited to subsets of ecstasy users [heavy users (Halpern, *et al*., 2004; Moeller, *et al*., 2002b), males (von Geusau, *et al*., 2004)] or have not reached statistically significance at conventional thresholds (Quednow, *et al*., 2007). In addition, the positive results to date have widely assumed that the impulsivity emerged as a consequence of ecstasy use via serotonergic neurotoxicity, but have not satisfactorily excluded the possibility that impulsivity pre-dates drug-taking, associated with vulnerability mechanisms (Lyvers, 2006). At a conceptual level, the 5-HT theory of impulsivity may represent an over-simplification given evidence from experimental animals that impulsive responses are negatively related to 5-HT levels in subcortical regions, but positively related to 5-HT efflux in the prefrontal cortex (Dalley, *et al*., 2002). These effects may cancel out following global serotonergic depletion in humans, and we previously reported no effects of dietary tryptophan depletion on response inhibition in healthy volunteers (Clark, *et al*., 2005).

A further problem lies in the measurement of impulsivity, which is increasingly viewed as a multi-factorial construct (Evenden, 1999b; Reynolds, *et al*., 2006). Substance abuse may be differentially associated with various components of impulsivity. Self-report impulsivity on questionnaire measures is elevated in substance users of various drugs (Moeller, *et al*., 2002a; Sher and Trull, 1994). These groups are also impaired on different laboratory tests of impulsivity, including delay discounting and response inhibition (Bickel and Marsch, 2001; Bjork, *et al*., 2004; Fillmore and Rush, 2002). In the delay discounting paradigm, impulsivity is defined as preference for immediate small rewards over larger delayed rewards. On tests of response inhibition, impulsivity is defined as a failure to suppress automatic or dominant responses. It is striking that whilst drug users display impairments on each of these measures, these different aspects of impulsivity are typically only weakly correlated with one another (Dom, *et al*., 2007; Reynolds, *et al*., 2006).

The present report focussed on a further aspect of impulsivity, which has received less attention in the context of drug use. ‘Reflection’ impulsivity refers to the tendency to gather and evaluate information prior to decision-making, where impulsivity is associated with a failure of reflective processing. The construct has typically been measured in children using the MFFT (Kagan, 1966), where the subject is presented with a template picture (e.g. a bicycle) and six similar variants. One variant is identical to the template, and must be identified on each trial. Impulsivity is indicated by rapid, inaccurate decisions. Two experiments in ecstasy users by Morgan (1998) reported reduced MFFT accuracy without significant effects on MFFT latency. However, the MFFT places high demands on visual search, visual working memory and strategy use, and these domains may be independently disrupted in recreational ecstasy users (Fox, *et al*., 2002), perhaps leading to inflated error rates (see Block, *et al*. (1974) and Clark, *et al*. (2006) for further critique of the MFFT).

In the present study, we have used an alternative measure of reflection impulsivity that was designed to circumvent several limitations of the MFFT. In the Information Sampling Test (IST), the subject is presented with a 5 × 5 matrix that conceals boxes that are each one of two colours (e.g. red or blue). The subject must decide which of the two colours lies in the majority under the matrix, by uncovering boxes one at a time. Once uncovered, boxes remain visible for the remainder of the trial such that the working memory load is negligible. As well as reducing visual search and working memory demand, the IST also enables the extraction of a direct measure of information sampling (the probability of being correct at the point of decision) rather than a speed-accuracy composite as in the MFFT. Critically, the extent of information sampling on the task is closely correlated with the number of incorrect judgments, meeting a core criterion for a test of reflection impulsivity (Evenden, 1999a). We have also shown previously that information sampling on the IST was associated with slow, accurate responding on the MFFT, providing evidence for concurrent validity (Clark, *et al*., 2003). Recently, we reported reduced information sampling in chronic amphetamine and opiate users (Clark, *et al*., 2006). Whilst healthy controls responded at 81% certainty (95% confidence intervals: 77–85%) when there was no cost to sampling information, current and former users of amphetamines or opiates tolerated significantly lower levels of certainty in their decision-making (Clark, *et al*., 2006). The present study aimed to extend these data by examining current and former ecstasy users, as well as drug-naïve controls, and a fourth group of regular cannabis users who did not report ecstasy use, as an active control group. We hypothesized that information sampling would be reduced in the cannabis users, and that this impulsivity would be further exacerbated in the current and former ecstasy users as a result of serotonin neurotoxicity.

## Materials and methods

### Subjects

Participants were 46 current ecstasy users, 14 former ecstasy users, 15 current cannabis users and 19 drug-naïve controls, who were recruited from newspaper and magazine advertisements in the Cambridge area. All ecstasy users reported a minimum of 30 separate uses of the drug. Current users reported abstinence for at least 3 weeks to allow for short-term recovery of serotonin function, and the Ex-ecstasy users reported abstinence for at least 1 year. No participant tested positive for recent stimulant use, as assessed by a blood screen. Demographic characteristics are displayed in Table 1. All participants completed the National Adult Reading Test (NART) (Nelson and Willison, 1991) as an estimate of verbal IQ, the Beck Depression Inventory to record abnormal mood symptoms and the Eysenck Impulsiveness-Venturesomeness-Empathy (IVE) questionnaire (Eysenck and Eysenck, 1991) to measure self-reported impulsivity. The protocol was approved by the Cambridge Local Research Ethics Committee (LREC number 02/076) and all volunteers provided written informed consent prior to participation.

**Table 1:** Demographic and personality variables of the four groups

	Drug-naïve	Ecstasy	Ex-ecstasy	Cannabis

*N*	19	46	14	15
Age	24.0 (3.6)	24.2 (6.7)	27.9 (6.6)	22.3 (4.3)
Gender (M:F)	12:7	33:13	6:8	5:10
Verbal IQ	114.6 (4.6)	110.4 (7.6)	110.3 (8.8)	111.3 (9.5)
BDI	3.5 (2.3)	9.3 (7.6)^a^	11.6 (9.2)^a^	5.3 (3.9)
IVE–Imp	6.8 (3.9)	10.6 (4.2)^a^	11.9 (5.1)^a^	8.9 (4.2)
IVE–Vent	10.4 (3.2)	11.0 (3.0)	9.4 (3.9)	10.9 (3.8)
IVE–Emp	12.0 (2.7)	12.7 (2.8)	14.5 (3.6)	13.7 (2.9)

M:F, male:female; BDI, Beck Depression Inventory; IVE, Eysenck Impulsiveness (Imp)-Venturesomeness (Vent)-Empathy (Emp) Questionnaire. ^a^*P* < 0.05 vs. Drug-naïve controls.

### The information sampling task

The task was administered on a touch-sensitive 10.5 inch monitor. Subjects completed a single practice trial, followed by 10 trials in each of two conditions: the Fixed Reward (FR) condition and the Reward Conflict (RC) condition. Condition order was counter-balanced across subjects. On each trial, subjects were presented with a 5 × 5 matrix of grey boxes, with two larger coloured panels at the foot of the screen. Touching a grey box caused the box to open (immediately) to reveal one of the two colours at the foot of the screen. The subject was asked to decide which colour was in the majority of the 25 boxes. They were told ‘It is entirely up to you how many boxes you open before making your decision’ [for complete instructions, see Clark, *et al.* (2006)]. To indicate their decision, the subject touched the corresponding panel at the foot of the screen, whereupon the remaining boxes were uncovered and a feedback message ‘Correct! You have won [x] points’ or ‘Wrong! You have lost 100 points’ was presented immediately, for 2 seconds. In the FR condition, the subject was awarded 100 points for a correct response, irrespective of the number of boxes opened. In the RC condition, 250 points were available to win at the start of the trial, which decreased by 10 points with each box opened, thereby creating a conflict between the level of certainty and the reward available. Incorrect responses yielded 100 points deduction in either condition. In both conditions, the inter-trial interval (ITI) was of variable delay (minimum 1s) such that the minimum interval between trial onsets was 30 s (e.g. if the trial was completed in 20 s, the ITI was 10 s). This feature was inserted to counteract impulsive behaviour due to delay aversion.

Performance was indexed by the average number of boxes opened, but in addition, the probability of making a correct choice at the point of decision was calculated on each trial [*P*(Correct); see Clark, *et al*. (2006) for formula]. Whilst these two variables are typically correlated with one another, under some circumstances the number of boxes opened can be a limited index of the information available; for example, 20 boxes may be distributed 10:10 [*P*(Correct) = 0.50] or 15:5 [*P*(Correct) = 1.0]. Consequently, the *P*(Correct) variable is related more directly to the levels of certainty tolerated during decision-making, and was therefore the primary variable for analysis. The number of errors was also recorded to test the impact of reduced information sampling on decision-making accuracy.

### Statistical analysis

Data were analysed with SPSS (SPSS Inc, Chicago, Illinois, USA) version 14 using two-tailed parametric tests thresholded at *P* < 0.05. Demographic and questionnaire data were analysed using one-way ANOVA and chi-squared tests as appropriate. The drug and alcohol use data were analysed with one-way ANOVA where normality assumptions were met, but in the most part, were not normally distributed and were analysed with nonparametric tests (Mann–Whitney and Kruskal–Wallis tests). IST performance was analysed using mixed-model ANOVA. Significant ANOVA group differences were decomposed using Tukey’s *post hoc* tests, or Tamhane’s T2 where variances were unequal.

## Results

### Demographic and drug use characteristics

The four groups did not differ significantly in NART-estimated verbal IQ (*F*_3,90_ = 1.49, *P* = 0.224), but the gender ratio differed significantly across groups (χ^2^ = 8.81, *P* = 0.031) (see Table 1). The ANOVA for group differences in ageapproached significance (*F*_3,90_ = 2.36, *P* = 0.077), due to slightly older age in the Ex-ecstasy group, although no *post hoc* tests were significant. There was a significant group difference in BDI score (*F*_3,90_ = 5.59, *P* = 0.001), due to elevated self-reported depression in the ecstasy and Ex-ecstasy groups in comparison to drug-naïve controls (Tamhane’s T2, *P* < 0.0001 and *P* = 0.038 respectively). Neither BDI score nor age was significantly correlated with IST performance (*r*_94_ = 0.013 and *r*_94_ = 0.177 respectively), so these variables were not considered as covariates. There was a significant group difference in self-reported impulsivity on the Eysenck IVE (*F*_3,90_ = 5.03, *P* = 0.003) due to elevated scores in the Current ecstasy and Ex-ecstasy groups in comparison to drug-naïve controls (Tukey’s, *P* = 0.007 and *P* = 0.006 respectively); the cannabis group did not differ from drug-naïve controls (*P* = 0.469). There were no group differences on the Venturesomeness (*F*_3,90_ = 0.866, *P* = 0.462) or Empathy (*F*_3,90_ = 2.46, *P* = 0.067) subscales.

Drug and alcohol use data are displayed in Table 2. All subjects consumed alcohol, although consumption (units/month) differed significantly (*F*_3,90_ = 5.8, *P* = 0.001) with the Current ecstasy group consuming more than that of the drug-naïve controls and cannabis users (Tamhane’s T2; *P* < 0.0001 and *P* = 0.001 respectively). All subjects in the three drug groups smoked cigarettes, with no differences in monthly consumption (*F*_2,72_ = 1.7, *P* = 0.191). The Current ecstasy and Ex-ecstasy groups were comparable in terms of lifetime ecstasy exposure (Mann–Whitney test; *Z* = 0.52, *P* = 0.606) and highest regular dosage (*Z* = 1.2, *P* = 0.219), but the Current ecstasy group reported higher peak single dose intake (i.e. the maximum number of tablets consumed on a single occasion) (*Z* = 2.6, *P* = 0.009), whereas the Ex-ecstasy group reported greater maximum frequency of usage per month (*Z* = 2.3, *P* = 0.019). As expected, the Ex-ecstasy group also had a longer abstinence period (*Z* = 5.6, *P* < 0.001). The cannabis users reported similar current cannabis usage (joints per month) to the two ecstasy groups (Kruskal–Wallis χ^2^ = 4.5, *P* = 0.106), although the two ecstasy groups reported more total lifetime usage of cannabis (χ^2^ = 6.1, *P* = 0.047). Subjects in the two ecstasy groups were more likely than the cannabis group to have ever used psilocybin (Fisher’s Exact χ^2^ = 19.1, *P* < 0.0001), LSD (χ^2^ = 16.2, *P* < 0.0001), amphetamine (χ^2^ = 21.2, *P* < 0.0001), amyl nitrate (χ^2^ = 21.8, *P* < 0.0001), ketamine (χ^2^ = 18.6, *P* < 0.0001), cocaine (χ^2^ = 17.9, *P* < 0.0001) and opiates (χ^2^ = 12.4, *P* = 0.001); although, there was modest usage of most of these substances in the cannabis group.

**Table 2:** Self-reported drug and alcohol use in the four groups [mean (SD)]

	Drug-naïve	Ecstasy	Ex-ecstasy	Cannabis

Alcohol (*N*)	19	46	14	15
Units last month	32.5 (28.1)	101.8 (99.5)	43 (66.8)	34.6 (25.1)
Tobacco (*N*)	0	46	14	15
Cigarettes last month	–	172.4 (191.5)	238.8 (239.3)	107.0 (140.9)
Cannabis (*N*)	9	46	14	15
Life joints	7.1 (4.5)	6707.7 (9244.1)	10 379.2 (18 546.5)	2704.2 (6221.4)
Joints last month	–	53.1 (80.9)	52.1 (121.9)	31.3 (53.7)
Ecstasy (*N*)	0	46	14	0
Life tablets	–	609.1 (703.2)	1000.8 (1792.4)	–
Peak intake (single dose)	–	8.9 (4.5)	5.3 (2.8)	–
Highest regular dose (tablets)	–	4.7 (2.5)	4.0 (2.8)	–
Highest regular frequency (times/month)	–	5.7 (3.8)	11.2 (8.0)	–
Time since last taken (days)	–	71.9 (66.0)	1059.4 (1105.8)	–
Psilocybin (*N*)	0	38	10	3
Times in lifetime	–	14.1 (20.5)	14.3 (23.4)	3.5 (2.2)
LSD (*N*)	0	31	11	2
Trips in lifetime	–	69.7 (165.2)	52.6 (115.3)	3.0 (1.4)
Amphetamine (*N*)	0	40	13	4
Grams in lifetime	–	401.8 (1361.3)	268.6 (371.8)	78.8 (155.5)
Amyl nitrate (*N*)	0	37	10	2
Times in lifetime	–	67.7 (255.8)	8.4 (6.8)	29.0 (26.9)
Ketamine (*N*)	0	27	5	0
Grams in lifetime	–	11.6 (18.8)	6.2 (6.9)	–
Cocaine (*N*)	0	41	14	6
Grams in lifetime	–	87.6 (165.8)	214 (687.5)	7.9 (9.3)
Opiates (*N*)	0	14	8	0
Grams in lifetime	–	9.2 (24.2)	76.8 (85.0)	–

### IST performance

**Figure 1:**
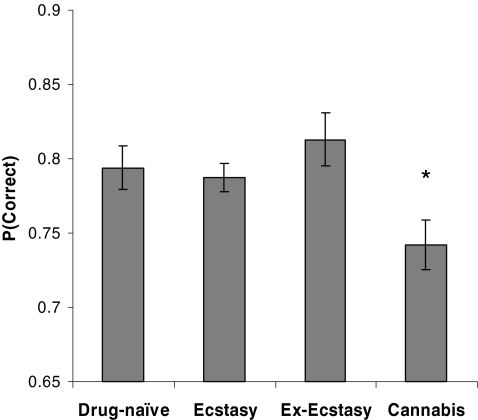
Performance on the Information Sampling Test in the Current and Ex-ecstasy users, cannabis users and drug-naïve controls, in terms of the probability of making a correct response at the time of decision [*P*(Correct)]. These data are collapsed across the two conditions of the task (Fixed Reward and Reward Conflict) given the absence of a significant group × condition interaction term. Error bars display standard error of the mean. The asterisk signifies *P* < 0.05 in the comparison against drug-naïve controls.

A mixed-model ANOVA of *P*(Correct) data (the probability of being correct at the point of decision), with Condition (Fixed Reward, Reward Conflict) as a within-subjects variable and Group and Gender as between-subjects variables, revealed a significant main effect of Condition (*F*_1,86_ = 95.8, *P* < 0.0001). As expected, subjects tolerated more uncertainty [a lower *P*(Correct)] in the Reward Conflict condition than the Fixed Reward condition, thus demonstrating sensitivity to the task contingencies (see Table 3). There was a significant main effect of Group (*F*_3,86_ = 5.45, *P* = 0.002), and a significant Group × Gender interaction (*F*_3,86_ = 4.53, *P* = 0.005). The other terms did not attain significance (all *F* < 1), and notably, the Group × Condition interaction was not significant (*F*_3,86_ = 0.621, *P* = 0.603) suggesting comparable sensitivity to the change in conditions across groups. *Post hoc* group comparisons (Tukey’s) collapsed across Condition showed that the cannabis users opened significantly fewer boxes compared with the Ex-ecstasy group (*P* = 0.013), and differed at trend from the Current ecstasy users (*P* = 0.076) and the drug-naïve controls (*P* = 0.078). There were no differences between the ecstasy groups and drug-naïve controls (see Figure 1). An *a priori* planned contrast confirmed a significant difference between the cannabis users and the drug-naive controls (*t*_32_ = 2.31, *P* = 0.027) with a large effect size (Cohen’s *d* *=* 0.81).

**Table 3:** Performance on the Information Sampling Task in the four groups [mean (SD)]

	Drug-naive	Ecstasy	Ex-ecstasy	Cannabis

Fixed reward				
*P*(Correct)	0.85 (0.10)	0.84 (0.09)	0.86 (0.08)	0.78 (0.07)
Boxes	14.8 (4.6)	15.4 (4.4)	15.7 (4.1)	11.8 (3.8)
Errors	1.0 (1.1)	1.3 (1.1)	1.1 (1.0)	1.8 (1.2)
Reward conflict				
*P*(Correct)	0.74 (0.06)	0.73 (0.06)	0.77 (0.07)	0.70 (0.07)
Boxes	8.9 (2.5)	9.3 (3.4)	11.2 (3.6)	8.2 (3.8)
Errors	2.5 (1.2)	2.3 (1.3)	2.2 (1.4)	2.1 (1.2)

A simple main effects analysis of the Group × Gender interaction assessed the effect of Group in males and females separately, collapsed across condition. The one-way ANOVA was significant for male subjects (*F*_3,52_ = 6.77, *P* = 0.001), where *post hoc* comparisons demonstrated significantly reduced information sampling in male cannabis users compared to each of the other three groups (Tukey’s: Ex-ecstasy users *P* < 0.001; Current ecstasy users *P* = 0.035; drug-naïve controls *P* = 0.038). The one-way ANOVA in female subjects was not significant (*F*_3,34_ = 1.46, *P* = 0.242), but numerically, the female cannabis group displayed the lowest information sampling of the four groups. A *post hoc* analysis compared the extent of cannabis usage (the main drug of abuse) across male and female subjects in the polydrug group, and found similar lifetime joints (*t*_4.1_ = 1.05, *P* = 0.350) and joints in the last month (*t*_4.5_ = 1.1, *P* = 0.324) in the male and female participants, suggesting that the influence of gender on the IST performance was not simply due to differences in drug usage.

Analysis of the number of boxes opened on the IST revealed a qualitatively similar pattern of group differences to *P*(Correct) data, which is unsurprising given *r* > 0.9 correlations between these variables (see task description in Methods). There was a significant main effect of group in the mixed model ANOVA (*F*_3,90_ = 5.83, *P* = 0.001) due to reduced information sampling in the cannabis users compared with the Ex-ecstasy group (Tukey’s *P* = 0.020) and the Current ecstasy group (*P* = 0.059). There were greater group differences in the male subjects (*F*_3,52_ = 7.07, *P* < 0.0001) than in the females (*F*_3,34_ = 1.83, *P* = 0.161). Errors committed on the IST was inversely correlated with boxes opened (*r*_94_ = –0.526, *P* < 0.0001) and *P*(Correct) (*r*_94_ = –0.566, *P* < 0.0001), confirming a core principle of reflection impulsivity. However, the mixed-model ANOVA of IST-errors found no significant main effect of Group (*F*_3,90_ = 0.258, *P* = 0.855) or Group × Condition interaction (*F*_3,90_ = 1.80, *P* = 0.152). Finally, we examined the correlation between Impulsivity and Venturesomeness scales of the Eysenck IVE, and IST performance [*P*(Correct) collapsed across condition]. There was no significant association in the overall group (Impulsivity: *r*_94_ = 0.003, *P* = 0.974; Venturesomeness *r*_94_ = 0.043, *P* = 0.678) or in any of the four groups (*r* = −0.33 to +0.19).

## Discussion

The present study used a recently developed IST to measure reflection impulsivity in current and former ecstasy users, cannabis users and drug-naïve controls. In the Fixed Reward condition (where there was no penalty for sampling further information), the drug-naïve control group sampled information to a point of 85% certainty (95% confidence intervals: 80–89%), similar to healthy performance in our previous study (Clark, *et al*., 2006). Moreover, the number of boxes opened and the level of certainty tolerated during decision-making were both inversely correlated with incorrect judgments in the overall sample (*n* = 94). This demonstrates the central feature of a test of reflection impulsivity: the extent of information sampling is predictive of eventual decision accuracy (Evenden, 1999a).

Regular cannabis users sampled significantly fewer boxes on the IST and tolerated more uncertainty in making the correct decision, compared with the other groups. This difference was statistically significant in a planned comparison against the drug-naïve control group, and the cannabis users sampled significantly less information than the Ex-ecstasy group in the more conservative Tukey’s *post hoc* group comparisons. The cannabis users altered their information sampling behaviour to a similar degree between the Fixed Reward and Reward Decrement conditions, compared with the other groups (i.e. the nonsignificant Group × Condition interaction term). This indicates comparable sensitivity to the change in reward contingencies, and suggests that the reduced information sampling behaviour was not simply attributable to a lack of motivation in the cannabis group. These data are also consistent with a report of risky decision-making (on the Iowa Gambling Task) in regular marijuana users (Whitlow, *et al*., 2004). Decision-making impairments on complex tests like the Iowa Gambling Task may putatively arise from a failure of pre-decisional information sampling or evaluation. Our findings extend our earlier observation of reduced information sampling in current and former users of amphetamines or opiates, who met DSM-IV criteria for dependence (Clark, *et al*., 2006). Given the presence of this effect across multiple substances of abuse with distinct pharmacological targets (amphetamines, opiates, cannabis), we suggest that impaired reflection impulsivity may represent a cognitive style associated with the pre-existing vulnerability to recreational drug use and later dependence, consistent with data from high-risk prospective studies (Nigg, *et al*., 2006; Tarter, *et al.*, 2004).

We were unable to detect any significant group differences on the IST between the ecstasy-using groups and the drug-naïve controls. Several other studies have failed to substantiate the link between ecstasy use and other aspects of impulsivity, including the Stroop and Go/No Go tests (Dafters, 2006; Fox, *et al*., 2002; Gouzoulis-Mayfrank, *et al*., 2003). However, our findings fail to replicate several studies that have demonstrated impulsivity in regular ecstasy users on another widely used test of reflection, the MFFT (Morgan, 1998; Morgan, *et al.*, 2002; Morgan, *et al*., 2006; Quednow, *et al*., 2007). The ecstasy users in the present study reported moderate use of other illicit substances, including similar cannabis usage to the cannabis group. The two ecstasy groups were also more likely than the cannabis group to have used a range of other substances, including amphetamine, cocaine and opiates. Consequently, if reduced reflection is a pre-existing cognitive style associated with general recreational drug use, we would expect this effect to have also been present in the two ecstasy groups. Lack of statistical power seems unlikely to explain the negative result, as the ecstasy groups actually sampled more information (in terms of boxes opened), on average, than the drug-naïve controls. In addition, the group size of 46 current ecstasy users is reasonably large for studies of this kind, and the level of ecstasy consumption was considerable (e.g. lifetime usage means of 609 and 1001 in the Current and Ex-ecstasy groups respectively), compared with the wider neuropsychological literature [e.g. 458 tablets in Quednow, *et al.* (2007)].

There are several possible explanations for the discrepancy with the studies by Morgan, *et al.* (1998, 2002, 2006), and Quednow, *et al*. (2007). One consideration is the duration of abstinence from ecstasy, which was relatively long in the present study (> 3 weeks) but much shorter in the positive studies, ranging from 3 days (mean 17 days; Quednow, *et al*., 2007) to 5 days (Morgan, *et al*., 2005). Studies in experimental animals reveal recoverable reductions in serotonin function 1–2 weeks after dosing that do not indicate neurotoxicity (Gouzoulis-Mayfrank & Daumann 2006). In addition, studies of other drugs indicate that short-term withdrawal may exacerbate behavioural impulsivity (see below). Lyvers and Hasking (2004) recommended a 1-month abstinence window for neuropsychological studies. Hence, the positive MFFT results by Morgan, *et al.* and Quednow, *et al.* could be caused by semi-acute effects of serotonin depletion upon task performance.

We have shown previously that IST performance is related to MFFT performance in healthy volunteers: fast, inaccurate responders on the MFFT opened significantly fewer boxes on the IST than slow, accurate responders (Clark, *et al.*, 2003). However, the MFFT involves a number of extraneous additional processes, including visual search, visual working memory and strategy implementation, which may be independently impaired in regular ecstasy users (Fox, *et al*., 2002; Halpern, *et al*., 2004; Wareing, *et al*., 2005). The design of the IST explicitly aimed to minimize these extraneous demands. In the MFFT study by Morgan (1998), there was a group difference in MFFT accuracy but not latency, which may be plausibly explained as a more general impairment. Other studies, however, reported significant differences in both speed and accuracy (Morgan, *et al*., 2006; Morgan, *et al*., 2002), which is likely to indicate impulsivity.

Additional factors may mediate the deficits in laboratory impulsivity in ecstasy users, and contribute to variability across studies. Gender may be one such variable: in the present study, the reduced information sampling in the cannabis group was mainly attributable to the male subjects, and other studies also described greater neuropsychological impairments in male drug users than in female drug users (Ersche, *et al*., 2006; Stout, *et al*., 2005), including ecstasy users (von Geusau, *et al*., 2004). In addition to gender, studies of other groups of drug-users with the delay discounting paradigm have indicated greater impulsivity in current users compared with ex-users (Bickel, *et al*., 1999; Petry, 2001). Two distinct mechanisms may contribute to this effect: withdrawal and/or craving may exacerbate impulsivity in current users (Field, *et al*., 2006; Giordano, *et al*., 2002), but also, less impulsive drug users may be more capable of achieving successful abstinence (Bickel, *et al*., 1999). In the present data, there was no evidence of the latter effect, as the Current and Ex-ecstasy users scored similarly on the IVE and IST measures. It is possible that the periods of abstinence from ecstasy in the Current (>3 weeks) and Ex (>1 year) ecstasy groups had attenuated impulsivity compared with the cannabis group, although against this explanation, the ecstasy groups did report moderate recent usage of other substances, including similar cannabis usage to the cannabis group in the past month.

Whilst we found no evidence of laboratory impulsivity on the IST in the ecstasy group, self-reported impulsivity on the Eysenck IVE questionnaire was significantly elevated in the current and former ecstasy users. Questionnaire impulsivity should indicate trait dispositions that are present prior to the initiation of drug use; for example, de Win, *et al*. (2006) showed no change on the Barratt Impulsiveness Scale before and after initiation of ecstasy use in a prospective cohort. Previous studies suggest large variability in trait impulsivity in ecstasy users, with a number of studies reporting elevations (Butler and Montgomery, 2004; Morgan, 1998; Parrott, *et al*., 2000), but other studies finding no differences (Travers and Lyvers, 2005) and one study even finding a significant reduction (McCann, *et al*., 1994). In our data, there was no association between the IVE score and performance on the IST. These data highlight the multi-factorial nature of impulsivity, and are in keeping with a number of other reports showing limited associations between state (laboratory) and trait (questionnaire) measures of impulsivity (Dom, *et al*., 2007; Lijffijt, *et al*., 2004; Reynolds, *et al*., 2006). Questionnaire ratings indicate general behavioural tendencies across a variety of situations, and rely on a subjective perception of one’s behaviour. In contrast, laboratory tasks provide an objective measure of a specific facet of impulsivity at a single point in time. Weak correlations between these two sets of variables may be a realistic expectation. Similarly, our findings do not refute the possibility that other domains of laboratory impulsivity (e.g. delay discounting, response inhibition) may be impaired in ecstasy groups. As discussed above, there are inconsistent findings using tasks of response inhibition in ecstasy users (Dafters, 2006; Fox, *et al*., 2002; Gouzoulis-Mayfrank, *et al*., 2003), and to our knowledge, no studies have yet explored delay-discounting in regular ecstasy users.

Some further limitations of the present study should be noted. Whilst the number of current ecstasy users was large, the group sizes for the former ecstasy users and the cannabis users were considerably smaller. In particular, the analyses split by gender should be treated as preliminary due to the reduced power, and need to be confirmed in a larger sample. In addition, the two groups of ecstasy users showed a high degree of polydrug use, although this arguably renders their intact IST performance even more surprising.

In conclusion, these data support the position of reflection impulsivity as a relevant cognitive dimension in regular drug users, by demonstrating reduced information sampling in a group of regular cannabis users. Reduced reflection is likely to have a detrimental impact on wider-scale decision-making capabilities, with potential relevance for treatment engagement and the ability to maintain long-term abstinence. Unexpectedly, the present study found no differences in reflection in current or former ecstasy users, despite evidence of trait impulsivity in these subjects. These data appear to challenge a simplistic pathway from ecstasy consumption to elevated impulsivity via serotonin neurotoxicity.
